# Supplementary material for: Comparative Analysis of Cytokine Expression Profiles in Prostate Cancer Patients
Source: Biology (Basel). 2025 May 6;14(5):505. doi: 10.3390/biology14050505 (PMC12109277; doi:10.3390/biology14050505)
Supplement: Supplementary file 1 [file biology-14-00505-s001.zip › biology-3588013-supplementary.pdf]

Comparative Analysis of Cytokine Expression Profiles in Prostate Cancer Patients

Karoline Brito Caetano Andrade Coelho [(1)], Denise Kusma Wosniaki [(2)], Jonatas Luiz Pereira [(3)], Murilo Luz [(3)], Letusa Albrecht [(2)], Jeanine Marie Nardin [(4)], Mateus Nobrega Aoki [(2)], Leonardo O. Reis *[(5,6,7)], Rodolfo Borges dos Reis [(1,7)] and Dalila Lucíola Zanette *[(1,2,7)]

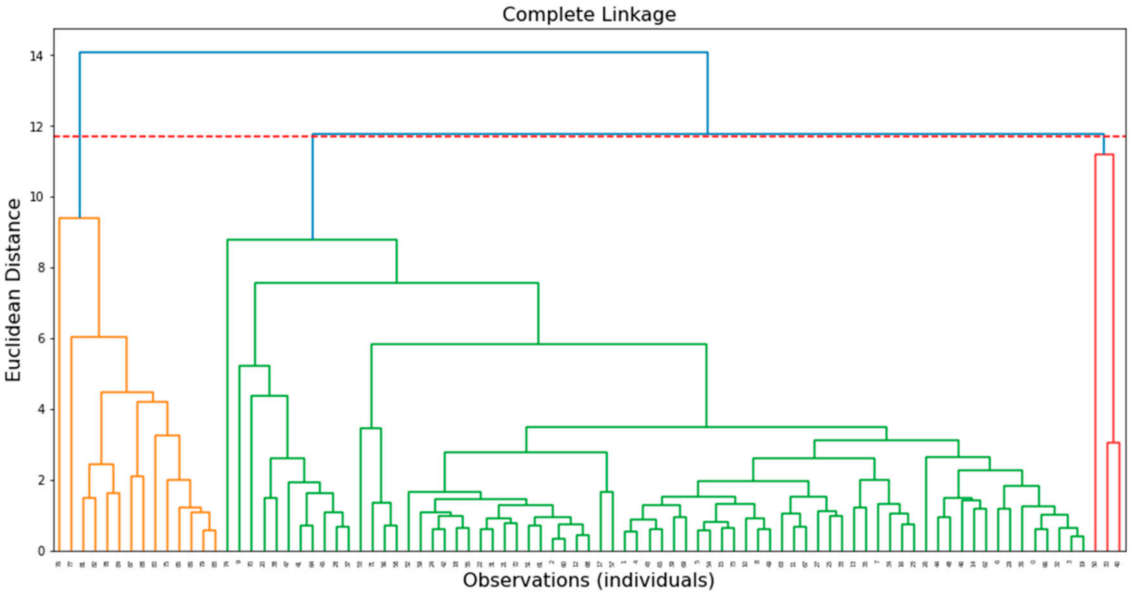

Supplementary Figure S1. Optimal number of clusters used as input for K-Means Clustering

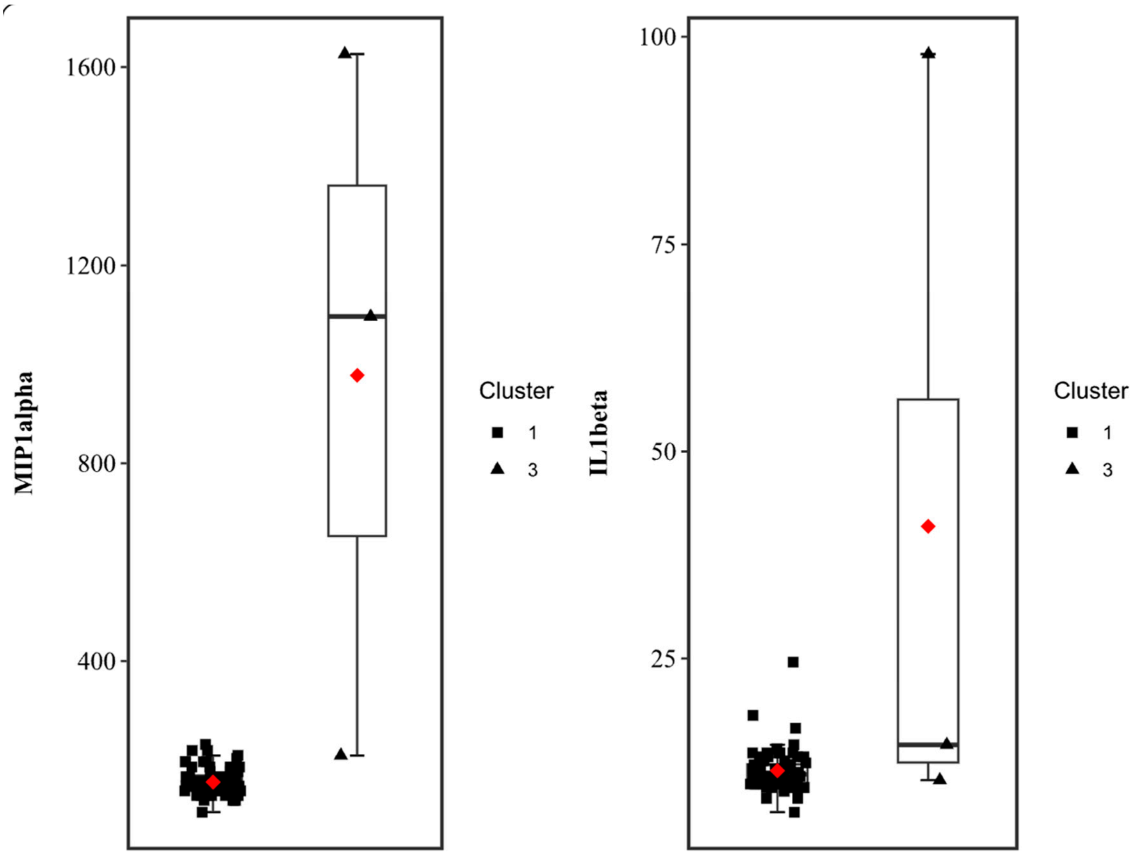

Supplementary Figure S2. Boxplot atypical expression levels (pg/dL) (Cluster 1 PCa patients) of two cytokines, IL1-beta and MIP1-alpha, compared to the rest of the cohort (Cluster 3 PCa patients).

Supplementary Table S1. Cytokine expression levels analyzed in the groups of patients stratified by 1A: ISUP grade (grades 1–3 vs. 4–5); 1B: prostate-specific antigen (PSA; <10 ng/mL vs. ≥10 ng/mL); and 1C: TNM stage (T2 M0/N0 vs. M1/N1/T3/T4).

1A) **Cyto-kine**	**group1** N = 24[1] (T2 M0/N0)	**group2** N = 22[1] (M1 / N1 / T3 /T4)	**p-value**[2]
MCP1	211 (131, 291)	203 (108, 296)	0.7
MIP1alpha	148 (139, 158)	158 (139, 178)	0.2
MIP1beta	240 (225, 267)	230 (210, 270)	0.6
IFNgamma	32.5 (28.8, 34.8)	31.0 (28.8, 36.3)	0.8
IL10	2.84 (2.16, 3.40)	2.70 (2.12, 3.54)	0.7
IL12p70	119 (100, 124)	110 (102, 115)	0.5
IL4	42 (32, 50)	38 (32, 42)	0.5
IL5	6.89 (5.96, 7.155)	6.76 (6.5, 7.02)	0.7
IL6	3.64 (2.88, 4.27)	3.38 (3.12, 4.14)	0.9
TNFalpha	6.91 (5.24, 8.34)	6.92 (6.30, 7.54)	0.5
IL1beta	10.84 (10.265, 12.28)	10.84 (9.92, 12.825)	>0.9
IL17	8.67 (8.26, 10.005)	9.08 (8.26, 9.9)	0.7

[1] Median, pg/dL (Q1, Q3). [2] Wilcoxon rank sum test.

1B) **Cytokine**	**PSA<10** N = 36[1]	**PSA>=10** N = 38[1]	**p-value**[2]
MCP1	206 (126, 327)	218 (125, 278)	0.9
MIP1alpha	153 (139, 182)	148 (139, 167)	0.4
MIP1beta	250 (230, 288)	230 (210, 256)	0.009
IFNgamma	31.8 (28.8, 36.3)	31.0 (28.8, 33.3)	0.4
IL10	2.93 (2.12, 3.70)	2.80 (2.20, 3.64)	>0.9
IL12p70	110 (98, 126)	108 (102, 115)	0.4
IL4	42 (34, 50)	38 (32, 50)	0.4
IL5	7.02 (6.50, 7.56)	6.50 (6.50, 7.02)	0.2
IL6	3.88 (3.38, 4.80)	3.88 (3.12, 4.40)	0.4
TNFalpha	7.22 (6.30, 8.02)	6.60 (5.68, 7.86)	0.2
IL1beta	11.76 (9.92, 13.42)	10.84 (9.92, 11.76)	0.2
IL17	9.08 (8.26, 9.90)	8.26 (8.26, 9.90)	0.6

[1] Median, pg/dL (Q1, Q3). [2] Wilcoxon rank sum test.

1C) **Cytokine**	**ISUP1_3** N = 47[1]	**ISUP4_5** N = 28[1]	**p-value**[2]
MCP1	218 (106, 296)	212 (158, 286)	0.6
MIP1alpha	158 (139, 187)	148 (139, 167)	0.14
MIP1beta	240 (220, 288)	230 (220, 253)	0.2
IFNgamma	31.8 (28.8, 36.3)	30.3 (28.8, 34.4)	0.4
IL10	2.70 (1.90, 3.74)	2.93 (2.36, 3.54)	0.4
IL12p70	110 (98, 124)	110 (101, 117)	0.8
IL4	42 (32, 50)	40 (32, 50)	0.6
IL5	6.50 (6.50, 7.56)	6.50 (6.50, 7.02)	>0.9
IL6	3.88 (3.38, 4.66)	3.64 (3.12, 4.80)	0.9
TNFalpha	7.22 (5.98, 7.86)	6.60 (6.14, 7.70)	0.7
IL1beta	11.30 (10.38, 13.18)	10.84 (9.92, 11.99)	0.2
IL17	8.26 (8.26, 9.90)	9.08 (8.26, 9.49)	>0.9

[1] Median, pg/dL (Q1, Q3). [2] Wilcoxon rank sum test.

Supplementary Table S2. PCa patients and controls (n=89) grouped into three clusters (two clusters with PCa patients – Clusters 1 and 3 - and one cluster with the control group – Cluster 2), pg/dL.

ID	grupo	MCP1	MIP1alpha	MIP1beta	IFNgamma	IL10	IL12p70	IL4	IL5	IL6	TNFalpha	IL1beta	IL17	Cluster
1	PCa	106.16	1626.00	220.00	30.30	4.72	102.00	31.78	6.50	2.88	5.68	10.38	8.26	3
2	PCa	130.92	1096.00	270.00	33.28	1.00	114.82	38.00	7.56	3.88	8.18	14.62	8.26	3
3	PCa	66.72	210.00	220.00	30.30	1.90	93.76	31.78	6.50	4.14	5.68	97.90	9.08	3
4	ctrl_f	200.32	2.83	46.01	6.66	0.50	3.24	0.03	1.60	0.71	8.39	0.68	1.67	2
5	ctrl_f	442.33	4.48	108.18	12.94	49.61	47.18	0.14	1.60	4.27	17.69	0.55	3.69	2
6	ctrl_f	260.85	6.47	102.87	4.18	20.57	37.62	0.45	6.29	16.83	16.83	11.56	3.69	2
7	ctrl_f	270.88	3.50	30.77	8.64	0.96	4.48	0.23	1.73	1.22	16.83	1.44	2.32	2
8	ctrl_f	307.17	5.48	35.78	6.66	1.34	3.24	2.34	1.73	0.75	11.40	4.94	1.92	2
9	ctrl_f	390.07	7.05	59.16	16.41	0.57	2.71	15.20	1.87	18.61	11.56	0.98	2.05	2
10	ctrl_f	281.29	3.76	42.66	8.64	2.45	10.30	0.08	3.50	1.65	24.53	0.98	1.67	2
11	ctrl_f	442.33	5.79	60.67	8.64	0.32	5.20	0.23	1.73	0.95	24.23	1.99	2.46	2
12	ctrl_f	315.00	2.71	46.01	7.92	1.84	8.64	0.40	1.87	1.54	10.30	0.98	1.80	2
13	ctrl_f	425.95	1.56	29.26	2.79	0.96	2.27	0.02	1.87	0.86	18.61	0.43	5.20	2
14	ctrl_f	420.63	4.59	60.67	5.34	0.66	4.48	0.06	3.04	0.64	12.94	0.75	2.26	2
15	ctrl_f	447.93	2.37	36.69	7.92	0.37	10.30	0.03	1.09	0.71	11.90	1.06	1.92	2
16	ctrl_f	713.27	2.37	43.75	4.18	0.27	2.71	0.03	3.69	0.51	18.14	0.61	1.43	2
17	ctrl_f	678.28	1.51	30.77	2.79	0.75	3.24	0.02	1.47	0.57	11.56	0.55	1.80	2
18	PCa	250.00	177.62	256.00	30.30	4.60	114.82	38.00	7.02	3.88	6.92	11.30	9.90	1
19	PCa	98.12	157.72	210.00	33.28	3.36	106.22	31.78	6.50	2.40	5.98	10.84	8.26	1
20	PCa	103.06	138.78	200.00	28.82	3.96	89.72	28.60	5.96	2.40	5.38	9.48	7.46	1
21	PCa	252.00	157.72	250.00	33.28	3.16	123.64	46.76	6.76	3.38	7.22	13.18	9.90	1
22	PCa	95.30	167.16	210.00	36.34	3.44	110.48	38.00	7.02	3.38	6.30	11.30	8.26	1
23	PCa	206.00	138.78	220.00	31.78	1.82	97.84	38.00	5.96	3.38	6.92	9.92	9.90	1
24	PCa	157.34	148.26	230.00	37.92	0.74	123.64	54.56	7.56	3.88	7.86	13.18	12.50	1
25	PCa	458.00	138.78	230.00	36.34	4.50	99.92	50.44	7.02	4.40	7.22	12.22	9.08	1
26	PCa	254.00	148.26	230.00	31.78	4.28	102.00	41.96	6.50	4.66	7.86	11.76	8.26	1
27	PCa	177.56	186.70	438.00	72.82	13.70	158.68	50.44	8.10	15.04	13.56	14.14	11.62	1
28	PCa	151.52	138.78	230.00	34.80	3.96	119.20	41.96	7.02	3.64	7.54	11.30	8.26	1
29	PCa	278.00	157.72	210.00	36.34	2.88	110.48	41.96	7.02	3.88	5.10	10.84	8.26	1
30	PCa	79.26	95.70	210.00	28.82	1.64	97.84	31.78	6.50	3.12	5.68	9.48	7.46	1
31	PCa	254.00	157.72	220.00	33.28	0.52	119.20	50.44	8.10	3.88	8.18	11.30	9.08	1
32	PCa	91.72	148.26	288.00	28.82	2.12	123.64	59.06	6.50	3.38	6.60	12.70	9.90	1
33	PCa	220.00	138.78	210.00	31.78	0.60	93.76	38.00	6.50	3.88	7.22	9.92	8.26	1
34	PCa	334.00	157.72	240.00	33.28	3.54	123.64	41.96	7.56	3.38	7.54	10.38	9.08	1
35	PCa	354.00	129.26	190.84	28.82	2.88	102.00	26.14	6.50	3.12	5.38	9.04	7.46	1
36	PCa	133.54	129.26	240.00	30.30	2.98	97.84	28.60	7.02	2.88	8.50	9.92	8.26	1
37	PCa	296.00	148.26	240.00	33.28	3.54	123.64	46.76	6.50	3.88	6.92	12.22	9.48	1
38	PCa	254.00	220.00	446.00	39.50	2.70	114.82	66.60	7.02	6.86	10.14	13.66	9.90	1
39	PCa	202.00	138.78	200.00	27.38	2.98	102.00	26.14	5.96	2.88	7.86	9.48	7.46	1
40	PCa	240.00	129.26	200.00	27.38	2.36	93.76	31.78	5.96	2.64	6.30	9.92	7.46	1
41	PCa	382.00	167.16	230.00	36.34	3.50	119.20	43.46	7.02	5.74	7.86	11.76	8.26	1
42	PCa	173.76	129.26	190.84	28.82	4.16	114.82	31.78	6.50	3.12	6.60	9.92	8.26	1
43	PCa	194.30	138.78	240.00	31.78	3.06	110.48	28.60	6.50	3.12	3.94	10.84	9.08	1
44	PCa	105.12	197.54	296.00	28.82	3.16	166.02	54.56	7.56	9.48	6.92	11.76	7.46	1
45	PCa	191.00	129.26	240.00	28.82	2.70	123.64	41.96	5.42	2.88	5.38	11.30	8.26	1
46	PCa	358.00	197.54	270.00	44.36	4.38	141.96	74.98	8.62	4.94	9.80	13.66	12.50	1
47	PCa	250.00	186.70	250.00	39.50	4.38	137.30	50.44	8.10	4.14	7.22	12.22	11.18	1
48	PCa	181.42	119.72	210.00	27.38	2.70	89.72	31.78	5.96	2.16	5.10	9.92	6.70	1
49	PCa	260.00	157.72	230.00	34.04	2.80	123.64	50.44	7.02	3.64	8.50	10.84	9.90	1
50	PCa	296.00	157.72	230.00	30.30	3.96	106.22	38.00	5.96	4.14	3.10	10.38	8.26	1
51	PCa	382.00	157.72	250.00	41.10	2.98	128.14	46.76	7.56	3.88	8.50	9.48	7.46	1
52	PCa	218.00	186.70	256.00	33.28	3.06	114.82	54.56	7.56	6.86	11.14	11.76	8.26	1
53	PCa	286.00	148.26	250.00	31.78	4.06	119.20	38.00	7.56	4.14	9.14	12.22	11.62	1
54	PCa	376.00	197.54	270.00	39.50	3.74	141.96	77.94	9.16	5.20	9.64	12.46	11.62	1
55	PCa	218.00	210.00	354.00	33.28	3.86	93.76	63.98	6.50	3.88	8.50	11.30	9.90	1
56	PCa	120.88	186.70	296.00	30.30	1.76	106.22	38.00	6.50	3.38	7.54	10.84	8.26	1
57	PCa	320.00	177.62	306.00	36.34	3.12	132.70	63.98	7.56	4.66	8.18	13.66	9.90	1
58	PCa	125.82	148.26	210.00	30.30	2.20	114.82	31.78	6.50	3.88	7.54	10.84	8.26	1
59	PCa	85.90	138.78	230.00	29.56	3.64	106.22	31.78	6.50	2.88	6.60	10.38	9.08	1
60	PCa	124.56	129.26	240.00	33.28	2.28	119.20	50.44	7.02	8.86	5.68	11.76	10.76	1
61	PCa	276.00	177.62	278.00	41.10	3.96	146.66	69.30	8.62	5.48	9.48	13.18	10.32	1
62	PCa	79.26	157.72	264.00	33.28	2.98	89.72	63.98	7.56	4.94	7.22	12.22	9.08	1
63	PCa	220.00	167.16	354.00	34.80	2.44	149.04	74.98	9.16	6.02	5.98	12.70	11.62	1
64	PCa	128.36	148.26	288.00	34.80	2.20	123.64	50.44	7.02	4.40	4.50	11.30	10.32	1
65	PCa	266.00	148.26	230.00	30.30	3.26	110.48	41.96	7.02	3.88	6.60	9.48	8.26	1
66	PCa	71.80	129.26	210.00	25.96	2.12	91.72	26.14	5.96	6.86	5.98	9.48	7.46	1
67	PCa	53.14	119.72	230.00	21.84	2.70	106.22	26.14	4.88	3.38	6.92	6.52	8.26	1
68	PCa	950.00	119.72	250.00	24.56	2.44	110.48	38.00	6.50	11.70	6.60	10.38	9.08	1
69	PCa	186.14	148.26	230.00	28.82	1.54	93.76	33.96	6.50	3.12	6.92	9.48	9.08	1
70	PCa	130.92	138.78	230.00	27.38	3.86	106.22	33.96	7.02	4.40	7.86	9.92	7.46	1
71	PCa	490.00	204.00	318.00	31.78	4.12	102.00	41.96	6.50	4.66	7.70	18.16	9.08	1
72	PCa	466.00	148.26	264.00	21.84	0.44	97.84	33.96	5.42	2.64	7.22	13.66	8.26	1
73	PCa	470.00	177.62	296.00	31.78	2.12	97.84	41.96	5.96	3.38	7.22	16.62	8.26	1
74	PCa	224.00	148.26	230.00	24.56	2.36	106.22	26.14	7.02	3.38	6.60	9.92	9.08	1
75	PCa	101.04	129.26	206.00	28.82	4.06	89.72	23.78	5.96	2.88	5.98	10.38	7.46	1
76	PCa	78.58	148.26	200.00	28.82	2.80	106.22	28.60	5.96	4.94	7.54	8.18	7.46	1
77	PCa	105.12	167.16	340.00	33.28	1.28	97.84	50.44	6.50	4.66	7.54	10.84	10.76	1
78	PCa	218.00	148.26	264.00	41.10	0.44	97.84	41.96	6.50	3.12	5.38	12.70	8.66	1
79	PCa	396.00	186.70	296.00	34.80	1.62	123.64	63.98	7.56	3.88	8.18	13.18	10.76	1
80	PCa	69.14	148.26	230.00	31.78	2.20	110.48	38.00	7.02	3.12	7.54	10.84	9.08	1
81	PCa	296.00	167.16	240.00	30.30	3.16	110.48	41.96	7.02	4.14	5.68	11.76	9.90	1
82	PCa	296.00	138.78	250.00	36.34	1.00	119.20	46.76	7.02	3.64	4.80	10.84	8.26	1
83	PCa	108.30	119.72	210.00	28.82	2.70	102.00	31.78	6.50	2.64	5.38	9.92	8.26	1
84	PCa	164.74	138.78	250.00	28.82	2.36	93.76	41.96	5.96	3.38	6.30	8.18	7.46	1
85	PCa	614.00	197.54	566.00	39.50	2.28	132.70	77.94	9.16	16.92	8.50	13.66	10.76	1
86	PCa	598.00	232.00	306.00	25.96	1.02	110.48	46.76	5.96	3.64	6.60	24.58	8.26	1
87	PCa	151.52	148.26	220.00	27.38	1.28	81.82	23.78	5.96	3.12	6.60	9.92	7.46	1
88	PCa	286.00	138.78	220.00	30.30	0.34	102.00	40.56	6.50	3.64	6.30	10.84	9.08	1
89	PCa	370.00	220.00	288.00	28.82	2.88	102.00	33.96	6.50	59.38	5.98	14.62	9.08	1
